# Identification of Intensity Ratio Break Points from Photon Arrival Trajectories in Ratiometric Single Molecule Spectroscopy

**DOI:** 10.3390/ijms13067445

**Published:** 2012-06-18

**Authors:** Dieter Bingemann, Rachel M. Allen

**Affiliations:** 1 Department of Chemistry, Williams College, 47 Lab Campus Drive, Williamstown, MA 01267, USA; 2 San Francisco Estuary Institute, Oakland, CA 94621, USA; E-Mail: rmallen86@gmail.com

**Keywords:** single molecule spectroscopy, ratiometric detection, statistical analysis, glass dynamics

## Abstract

We describe a statistical method to analyze dual-channel photon arrival trajectories from single molecule spectroscopy model-free to identify break points in the intensity ratio. Photons are binned with a short bin size to calculate the logarithm of the intensity ratio for each bin. Stochastic photon counting noise leads to a near-normal distribution of this logarithm and the standard student *t*-test is used to find statistically significant changes in this quantity. In stochastic simulations we determine the significance threshold for the *t*-test’s *p*-value at a given level of confidence. We test the method’s sensitivity and accuracy indicating that the analysis reliably locates break points with significant changes in the intensity ratio with little or no error in realistic trajectories with large numbers of small change points, while still identifying a large fraction of the frequent break points with small intensity changes. Based on these results we present an approach to estimate confidence intervals for the identified break point locations and recommend a bin size to choose for the analysis. The method proves powerful and reliable in the analysis of simulated and actual data of single molecule reorientation in a glassy matrix.

## 1. Introduction

Single molecule experiments can probe structure and dynamics on a molecular scale, revealing details that in traditional bulk experiments remain hidden behind ensemble averages [1–8]. In particular, the technique allows the observation of individual events of molecular dynamics, yielding distributions of and correlations between different dynamical properties. Unfortunately, as the method is still young [5], few analysis methods are available to harvest the vast pool of information from the original fluorescence intensity or photon arrival trajectories. Single molecule experiments are therefore often analyzed with correlation functions building on the common practice for bulk experiments. Even though differences between individual molecules observed in the sample are preserved, many details embedded in the temporal sequences of events are lost in the correlation procedure, which time-averages over the trajectory. Furthermore, properly averaged autocorrelation functions require a trajectory length of at least 100-times the correlation time [9], while experimental trajectories are often much shorter than this minimum due to the limited photochemical lifetime of the probe molecules.

Recently, methods were introduced that permit the correlation analysis of photon arrival times directly without binning [10,11], which, while increasing the time resolution achievable, still suffer from the time-averaging intrinsic in correlation analysis. Starting with the analysis of quantum dot and single molecule “blinking”, a drop of the total intensity to zero [1,12–19], often associated with excursions to the triplet state of the single molecule [20,21], the advantage of the identification of individual events in the single molecule trajectory became obvious. Numerous methods that are based on model dynamics were subsequently introduced, such as the analysis of hidden Markov chains, for example through Bayesian analysis [22–24], photon counting histograms [25], or maximum likelihood analysis [26,27]. These approaches are appropriate for single molecule dynamics with a well-known number of accessible states, for example in the case of blinking or enzymatic turnovers, which can be approximated as “on” and “off” states, but even this simplification is debated [28].

Several model-independent methods are described in the literature, which are capable of detecting individual intensity change points directly from a single-channel photon arrival trajectory with Bayesian or maximum likelihood approaches [29–31], yielding the times of sudden changes in a piece-wise constant fluorescence intensity trace. These methods do not require the assumption of an underlying mechanism or a limited number of accessible states, and can therefore be applied more general. Typical applications are the identification of enzymatic turnovers [32], motor protein movements [33], or nanoparticle blinking [18,19,34], all leading to large fluctuations in the fluorescence intensity associated with the dynamical event of interest. Those sudden changes in the fluorescence are common in single molecule spectroscopy, caused by jumps between states with different emission characteristics as opposed to the continuous changes seen in bulk.

In addition to the single-channel intensity detection employed in the examples above, ratiometric measurements, that is the simultaneous recording of two intensity channels, is another widely used technique in single molecule spectroscopy [2,35,36]. Examples are the detection of two different polarization directions of the emitted fluorescence, *I*_||_ and *I*_⊥_, to observe the angular reorientation of a single probe molecule reporting on polymer [37–39] or protein dynamics [40] as well as the detection of two different emission wavelengths, either to observe shifts in an emission spectrum [41] or to determine the distance between a pair of single molecules showing Förster resonance energy transfer (*sp*-FRET) [42–44].

Instead of investigating the intensities directly, a ratiometric analysis focuses on a normalized intensity ratio, for example, in the case of single molecule orientational motion, the reduced linear dichroism [45] (Equation 1).

(1) $$I_{d}=\frac{I_{\left|\right|}-I_{⊥}}{I_{\left|\right|}+I_{⊥}}$$

Here, effects of the photodynamics of the probe molecule, which might change the total intensity, *I*_||_+ *I*_⊥_ without affecting the intensity ratio, *I*_||_*/I*_⊥_, are eliminated from the monitored quantity, *I_d_*, and only changes in the polarization direction of the emission, caused by single molecule reorientation, are recorded. It would be desirable to detect sudden changes in the ratiometric measure similar to their detection in the single-channel analysis methods discussed above. A two-state Markov-chain approach has for example been used to analyze *sp*-FRET experiments [44,46].

This intensity ratio is central to any of the two-channel experimental methods mentioned above. As an example for its usefulness we will here discuss one application, monitoring the fluorescence polarization direction of the single molecule emission. However, the method described here is general and can easily be adapted to any ratiometric single molecule technique.

**Figure 1:**
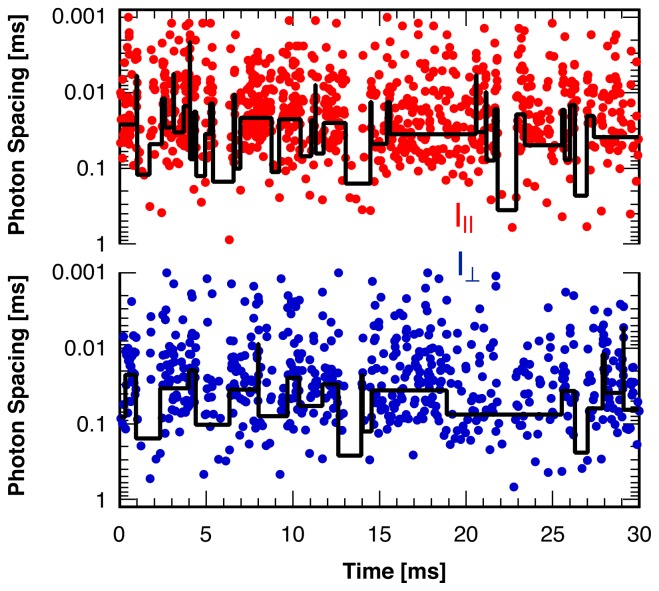
Waiting times between two consecutive photons recorded in two perpendicular polarization directions as a function of photon arrival time for the fluorescence of a single rhodamine B molecule in a solid polymer matrix. “Blinking” leads to frequent gaps (“dark” periods) in the stream of photons with durations on the order of a millisecond. Line: Result of the identification of intensity change points in each detection channel separately with a maximum likelihood method [29].

To construct a model-free approach for the detection of change points in a ratiometric variable one might imagine analyzing both intensity channels separately using one of the single-channel model-free methods [29–31] and combining the results. However, single probe molecule “blinking” that leaves the ratiometric measure of interest (such as the reduced linear dichroism, *I_d_*) undefined during any “dark” periods interferes with this approach. An example illustrating the high frequency and short durations of these blinking events is shown in Figure 1 for the two polarization directions of the fluorescence from a single rhodamine B molecule immobilized in a solid polymer matrix. Short gaps in the continuous stream of photons indicate frequent blinking events with typical durations on the order of a millisecond. Also shown are the results of a statistical analysis routine [29] analyzing the photon arrival times in each detection channel separately. This routine, in combination with a subsequent coincidence analysis [47] would need to identify all of these blinking periods to avoid false positive ratiometric change points, the quantity of interest in the applications mentioned above. As can be seen from Figure 1 this is clearly not the case, justifying the search for a new statistical analysis method dedicated to the identification of ratiometric change points, as presented in this paper.

## 2. Results and Discussion

### 2.1. Threshold Values for Significance

**Figure 2:**
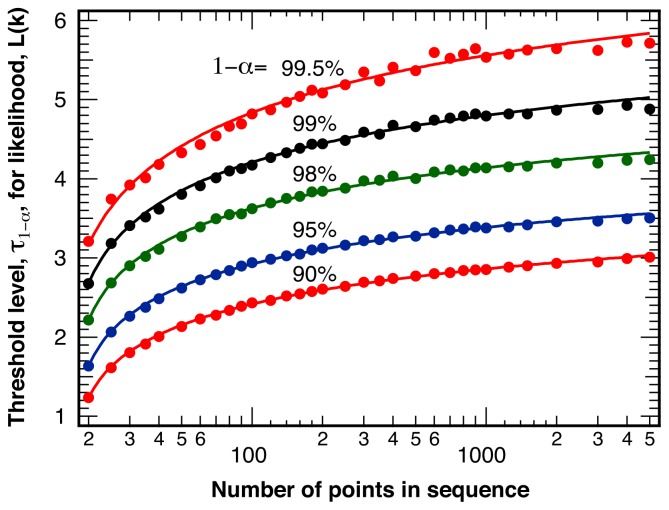
Threshold values, *τ*_1−_*_α_*, for the likelihood measure *ℒ*(*k*′) corresponding to statistically significant intensity ratio break points at a level of confidence of 1 − *α* as indicated. Points: results of *N* = 100,000 random number simulations with *〈N_photon_〉* = 25 photons per bin and *〈ρ〉* = 1. Lines: smooth fits with an empirical function (Equation 2).

The threshold value, *τ*_1−_*_α_*, indicating statistically significant differences between two sections of intensity ratio points with a total length *L* at various levels of confidence 1−*α*, is shown in Figure 2. The threshold value *τ*_1−_*_α_* does depend very weakly on both the average intensity ratio, *〈ρ〉*, between the two detection channels, and the average number of photons per bin, *〈N_photon_〉*, and is shown here only for equal intensity in the two channels, *〈ρ〉*= 1, and *〈N_photon_〉*= 25 photons per bin. We fit the slow increase of the threshold value *τ*_1−_*_α_* as a function of the total number of sample points, *L*, with the empirical fit function:

(2) $$\tau_{1-\alpha }(L)=A\cdot {\left[\text{log}\;(\text{log}(s\cdot L))\right]}^{\lambda }$$

where *A* represents an amplitude, *s* a scaling factor, and *λ* a power law exponent. Table 1 lists the resulting best-fit parameters for the various levels of confidence, 1−*α*, for the fits displayed in Figure 2.

If the analysis is performed with the additional safeguard that *N_min_* = 10 consecutive points have to surpass the threshold value, *τ*_1−_*_α_*, the effective probability, *α_eff_*, of false positives is further suppressed, for example to *α_eff_* = 0.05% in the case of *α* = 1%, as listed in Table 1. This safeguard might seem overly cautious, but in our particular application we are interested in an accurate identification of large intensity ratio changes, *ρ_i_/ρ_i_*_+1_, and the waiting times, *t_w_*, in between. Break points missed because of this additional safeguard are either characterized by small intensity ratio changes, *ρ_i_/ρ_i_*_+1_, or a short waiting time, *t_w_*, leading up to the break point (see Section 2.3), neither one of which constitutes a shortcoming in the analysis of our single molecule experiments [38].

**Figure 3:**
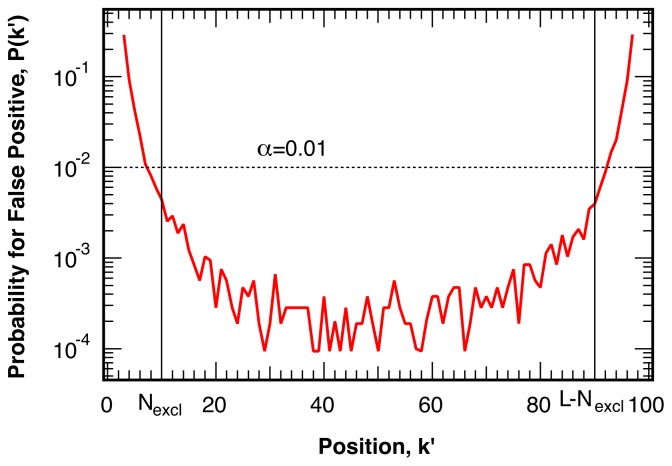
Probability for false positive break points as a function of the trial location *k*′ in simulated intensity ratio trajectories without a break. The ideal, uniform, distribution at a level of confidence of 1−*α* = 99% is indicated by the dotted line. The first and last *N_excl_* = 10 points, whose probability for false positives approaches or exceeds the ideal target value *α*, are excluded in the analysis.

Trial break points, *k*′, near the beginning (*k*′ *→* 0) or the end (*k*′ *→ L*) of the investigated sequence of the trajectory generate one section with a very small sample size. The resulting large fluctuations in the mean and standard deviation of this small section lead to an increased probability of false positive identification, as shown in Figure 3 for *N* = 100,000 simulations for sequences of length *L* = 100. The increased probability of false positives near one of the limits of the sequence does not depend on the length of the remainder of the sequence, *L* − *k*′. As this increased likelihood for the identification of false break points significantly exceeds the ideal, targeted, probability of *α*, as indicated by the dashed line in Figure 3, we exclude *N_excl_* = 10 points at the beginning and the end of the investigated sequence of intensity ratio points from the analysis.

### 2.2. Accuracy

**Figure 4:**
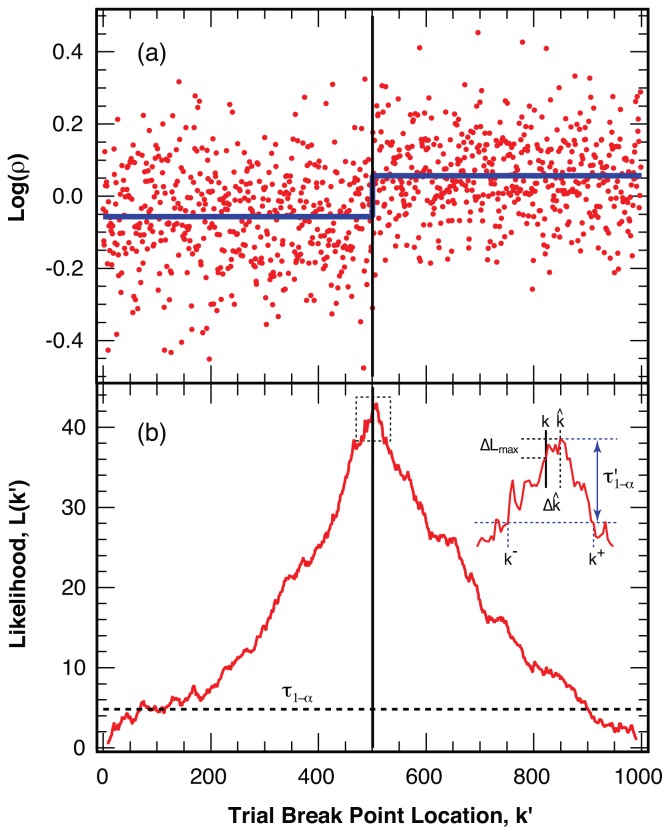
Identification of the most likely break point in a simulated example (**a**) Logarithm of the intensity ratio, log(*ρ*), with *〈ρ〉* = 1, for 1000 simulated points (red) with *〈N_photon_〉* = 25 photons per bin and a 30% change in the intensity ratio at the center point (thin vertical line). The average intensity is indicated with a thick line (blue); (**b**) Likelihood measure, *ℒ*(*k*′), for a break at all possible trial positions, *k*′, for the trajectory in panel (**a**). The threshold value, *τ*_1−_*_α_*, for a level of confidence 1 − *α* = 99% is indicated with the dashed line. The position, *k̂*, of the maximum likelihood, *ℒ_max_* = *ℒ*(*k̂*), is taken as the best estimate for the break location. Inset: Expanded view of the likelihood for a break around the maximum, illustrating the determination of the confidence interval *k*^−^ *. . . k*^+^, around *k̂* using the threshold value *τ*′_1−_*_α_* (blue).

To illustrate the strength of the analysis method, Figure 4a displays an example with *L* = 1000 simulated intensity ratio points with *〈N_photon_〉* = 25 photons per bin. The sequence features a break point in the center, *k* = *L/*2, with a change in the intensity ratio of 30%. The corresponding likelihood measure, *ℒ*(*k*′), calculated at each trial break point, *k*′, for this example is displayed in panel (b) of Figure 4. The maximum likelihood value, *ℒ_max_*, significantly exceeds the threshold value *τ*_1−_*_α_* (added as a dashed line for the 1 − *α* = 99% level of confidence) for this sample length, leading to a clear identification of the simulated break point. We use the location of the maximum likelihood value, *ℒ_max_* = *L*(*k̂*), as the best estimate, *k̂*, for the location of the actual break point, *k*.

#### 2.2.1. Distribution of Location Error

**Figure 5:**
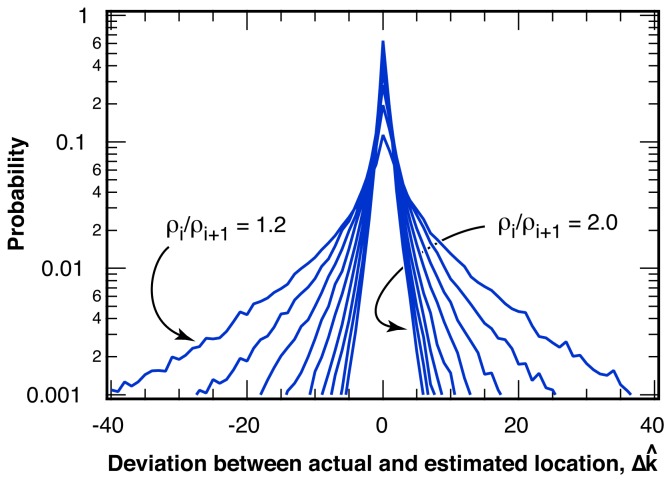
Distribution of the deviation between actual and estimated location of the break point, Δ*k̂* = *k̂* − *k*, for various changes in the intensity ratio at the break point, *ρ_i_/ρ_i_*_+1_, ranging from 1.2 to 2.0 in steps of 0.1, for an average number of *〈N_photon_〉* = 25 photons per bin.

**Figure 6:**
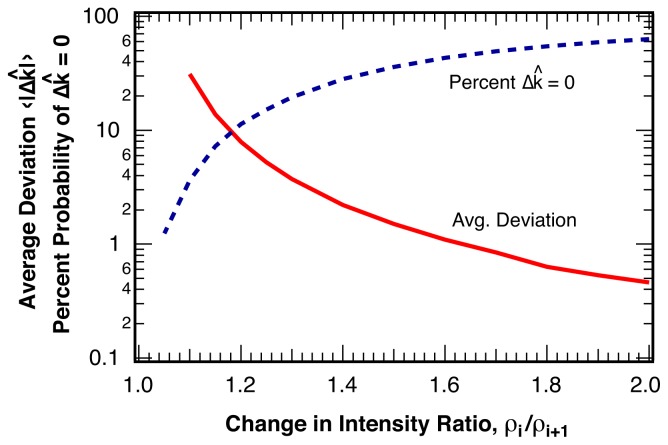
Solid line: Average error, *〈*|Δ*k̂*|*〉*, for the estimated location, *k̂*, of the break point, *k*, as a function of the change in the intensity ratio at the break point *ρ_i_/ρ_i_*_+1_ for *〈N_photon_〉* = 25 photons per bin. Dashed line: Percentage of correctly identified break point locations, *P*(Δ *k̂* = 0). We find no statistically significant dependence of the error on the length of the sample or on the position of the break point within the sample.

The distribution of the deviation, Δ*k̂* = *k̂*− *k*, of the estimated location for the intensity ratio break point, *k̂*, from the actual break point, *k*, is shown in Figure 5 for various changes in the intensity ratio, *ρ_i_/ρ_i_*_+1_. The probability *P*(Δ *k̂*) falls off approximately exponentially from a maximum probability at zero error, Δ *k̂* = 0 and only depends on the change in the intensity ratio, *ρ_i_/ρ_i_*_+1_, at the break point, but not on the length of the sample, *L*, or on the relative location, *k/L*, of the break point within the sample. For sequences similar to the example pictured in Figure 4 with *ρ_i_/ρ_i_*_+1_ = 1.30 and *〈N_photon_〉* = 25 photons per bin, over 20% of all break points are identified without error, Δ*k̂* = 0. The average deviation of all identified break points is *〈*|Δ*k̂*|*〉* = 3.7 bins. The average deviation, *〈*|Δ*k̂*|*〉* is shown in Figure 6 as a function of the relative change in intensity ratio, *ρ_i_/ρ_i_*_+1_, for a bin size of *〈N_photon_〉* = 25 photons per bin. Also indicated in Figure 6 is the percentage of correctly identified break point locations, *P*(Δ*k̂* = 0).

The selection of the bin size, *〈N_photon_〉*, determines the photon counting and single molecule blinking noise of the binned intensity ratios, the number of intensity ratio points to be analyzed between two break points, and the time resolution of the analysis. The distribution of the error of the estimated location, however, if measured in terms of the absolute deviation in time instead of as a number of bins, proved to be independent of the bin size chosen for the analysis of a given photon sequence. Binning fewer photons per bin might increase the time resolution per bin, but due to the increased photon counting noise per bin will not change the timing error in the estimated break point locations. The only way to reduce the timing error for break points even further is to record the photon trajectory at a higher intensity, thus increasing the number of photons available for analysis for the same number of break points.

#### 2.2.2. Estimation of Location Error

From *N* = 10,000 random number simulations with one break point in the center we find empirically that the threshold *τ*′_1−_*_α_*, which defines the confidence interval for *k̂*, depends strongly on the length, *L*, of the sequence tested, the average number of photons per bin, *〈N_photon_〉*, and the size of the step at the break point, *ρ_i_/ρ_i_*_+1_. However, for any combination of these three parameters investigated we find that the threshold value *τ*′_1−_*_α_* falls within 20% of a common upper bound, if described as a function of the variable *ℒ_max_/L*, where *ℒ_max_* is the likelihood of the estimated break point for a particular sequence of length, *L*. We describe this upper bound empirically through a power law that approaches a constant level for small values of *ℒ_max_/L*.

(3) $$\tau_{1-\alpha }^{\prime }\leq \tau_{0}^{\prime }+(1-\text{erf}\;(-\text{log}(x)))\cdot Ax^{\lambda }$$

where *x* = *ℒ_max_/L*, *τ*′_0_ is a constant threshold for small values of *ℒ_max_/L*, *A* signifies an amplitude, and *λ* is a power-law exponent. Table 2 lists the resulting parameters describing this upper bound for various levels of confidence, 1 − *α*. In tests on simulated photon trajectories of various lengths and step sizes we find that the fraction of the actual break points that lie within the thus estimated confidence interval is close to the expected value 1 − *α* for most parameter combinations. Only for very short sequences or few photons per bin do we observe the fraction of break points within the confidence interval to drop below 1 − *α*.

### 2.3. Sensitivity

**Figure 7:**
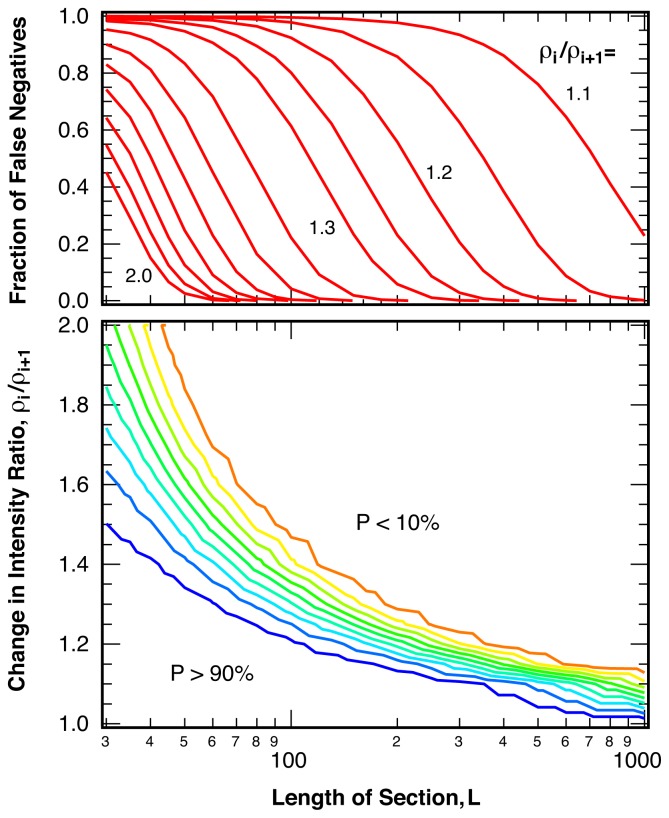
Probability of undetected break points (false negatives), *P*, as a function of the magnitude of the change in intensity ratio at the break point, *ρ_i_/ρ_i_*_+1_, and length of the test sequences, *L*, for sequences containing one break point in the center. *〈N_photon_〉* = 25 photons per bin and 1 − *α* = 99%. Contour lines are spaced in 10% intervals.

We determine the sensitivity of the analysis, that is the probability of false negatives, or break points missed, by testing the method described above on two types of sequences of simulated intensity ratio points. The first set of tests is performed on sequences that include a single break point at location *k*, the second set of tests uses simple model sequences with multiple break points (*N_jumps_* = 200) of identical spacing and constant changes in the intensity ratio (square waves). The probability of false negatives in sequences with a single break point depends strongly on the change in intensity ratio, *ρ_i_/ρ_i_*_+1_, the average number of photons per bin, *〈N_photon_〉*, and the length of the investigated sequence, *L*. Figure 7 shows these dependences for a bin size of *〈N_photon_〉* = 25 photons per bin both as a contour plot and as cuts for a variety of intensity ratio changes. As expected, closely spaced break points with small changes in the intensity ratio are likely missed, but both closely spaced large jumps as well as well-separated small jumps can be detected reliably.

The algorithm employed to search for multiple break points in photon arrival trajectories (Section 4.4) was separately tested on simulated model trajectories with multiple break points. The resulting probability for false negatives is very similar to the diagram depicted in Figure 7, except for a reduced sensitivity for break points in very short sequences (*L <* 40 points) caused by the additional safeguards added to protect against false positive identifications (Section 4.1).

**Figure 8:**
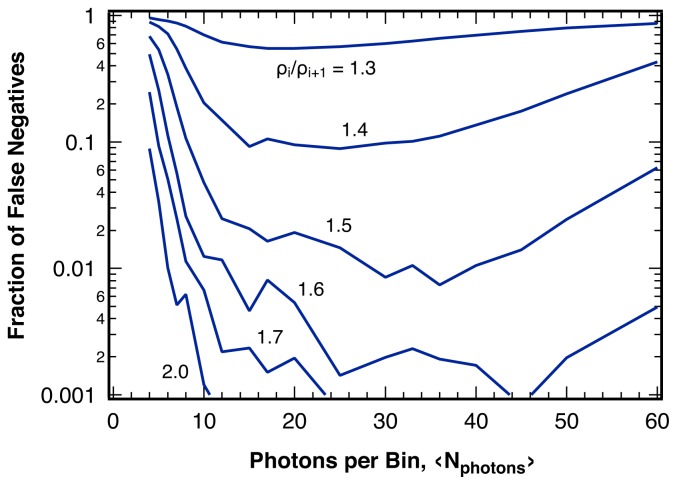
Fraction of undetected break points (false negatives) for various changes in the intensity ratio, *ρ_i_/ρ_i_*_+1_, as indicated, in sequences of photons with 2000 photons per break point as a function of the average bin size, *〈N_photon_〉*, for 1 − *α* = 99% using the analysis algorithm invoking the additional safeguards discussed in the text.

For a given sequence of photons, smaller bin sizes, *〈N_photon_〉*, lead to higher photon counting noise per bin for the intensity ratio, which, despite producing more bins to analyze for the sequence, reduces the sensitivity significantly (Figure 8). This observation is in contrast to results for statistical single-channel photon trajectory analysis, where the largest information content is revealed using a photon-by-photon approach [10]. On the other hand, large bin sizes can reduce the number of bins per break point below the additional safeguards introduced to guard against false positives (Section 4.1), such that actual break points could be rejected.

### 2.4. Comparison to Existing Methods

Returning to the challenge of analyzing single molecule photon arrival trajectories containing frequent blinking events (Figure 1) we now compare the results of the method described in this paper to those from the separate identification of intensity break points in each channel in combination with a subsequent coincidence analysis. A maximum likelihood analysis [29] of the photon arrival times in a single detection channel can identify many of the “dark” of “bright” periods, but due to their high frequency and very short duration nevertheless misses a significant fraction randomly in one of the two channels, as illustrated in Figure 1. A subsequent test for coincidences of these breaks points in both channels [47] to distinguish changes in the intensity ratio from changes in the total intensity therefore yields false-positive change points for the ratiometric measure whenever a break point is missed in one of the two channels.

**Figure 9:**
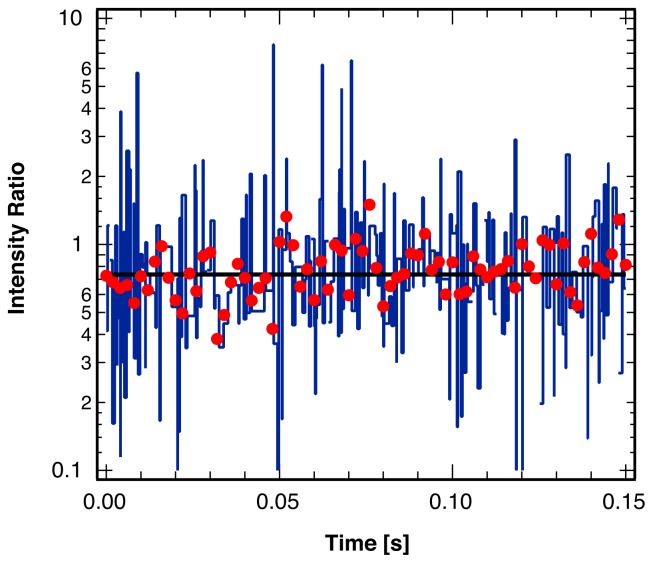
Ratio of the intensity of the two polarization components of the fluorescence from a single molecule embedded in a rigid polymer matrix, binned at 2 ms (red points). Result from the statistical analysis described in this paper (black line) indicating the expected lack of any reorientations of the probe molecule. Results from the separate identification of intensity break points in each polarization channel (blue line), frequently misidentifying blinking events as single molecule reorientations.

Figure 9 illustrates the result of this coincidence analysis for the photon arrival trajectory shown in Figure 1. As the trajectories were collected for a single molecule in a rigid polymer matrix, no molecular reorientations and therefore no changes in the fluorescence intensity ratio occur in the experiment. As shown, the single-channel analysis leads to a dramatic overestimation of the single molecule orientational dynamics. In comparison, the statistical analysis of the photon frequency, as suggested in this paper with a very short bin width on the order of the average blinking duration, yields the expected result of a constant ratio without any break points.

**Figure 10:**
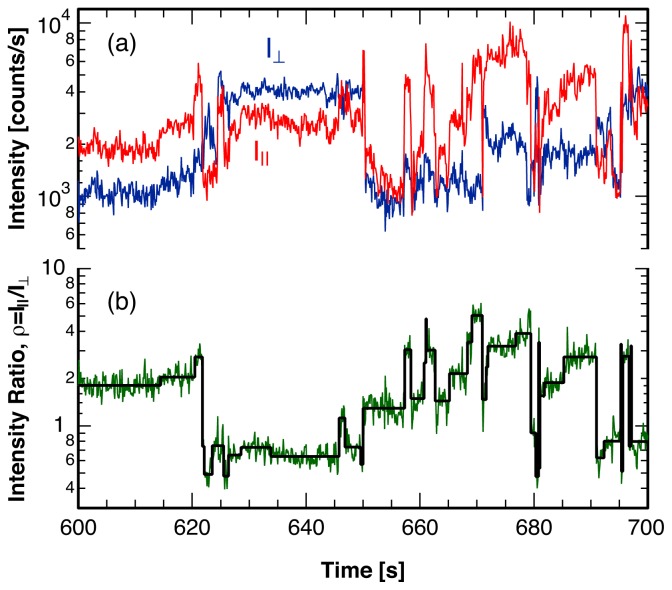
(**a**) Intensity of the two polarization components, *I*_||_ and *I_⊥_*, of the fluorescence emitted by a single rhodamine B molecule embedded in poly(vinyl acetate) at the glass transition temperature; (**b**) Ratio of the intensity of the two polarization components, *ρ* = *I*_||_*/I_⊥_*, (green) and sequence of single molecule angular jumps (black), reconstructed using the analysis method described in this paper.

As an example of the application of the proposed method to a single molecule trajectory with reorientations, Figure 10 illustrates the strength of the method by comparing an experimental trajectory with the corresponding reconstructed single molecule orientational dynamics. The reconstructed trajectory follows the experimental intensity ratio very well, capturing all significant reorientations of the probe molecule. Even more importantly, dynamical quantities such as the correlation function for the orientational motion of the probe molecule are represented equally well with the reconstructed trajectory [38]. In analogy to the discussion in the proceeding sections, we tested the accuracy and sensitivity of the analysis method applied to simulated single molecule trajectories where jump times and amplitudes were known. The simulated trajectories very closely resemble those recorded in single molecule experiments in a glass matrix at the glass transition temperature [38]. The dynamics in a glass is characterized by a very broad distribution of waiting times, spanning several orders of magnitude. In addition, the assumed exponential distribution of jump sizes generates a large number of break points with small changes of the intensity ratio. Even though both of these factors present a challenge to the analysis routine, the overall performance is very satisfactory, as basically all simulated break points with a change in the intensity ratio of *ρ_i_/ρ_i_*_+1_ *>* 1.25 are identified with 63% of the extracted jump times exhibiting an error in the location of less than 2 bins. This error in the location is well within the intrinsic timing error expected for single molecule experiments for a given recorded photon rate and can be controlled by choosing the excitation intensity appropriately.

## 3. Single Molecule Experiment

In single molecule spectroscopy, a fluorescent probe is embedded in the matrix of interest at a very low concentration [3–5]. We use rhodamine B as a probe molecule in the polymer poly(vinyl acetate) in the vicinity of the glass transition temperature of the matrix. Details of the experiment are published elsewhere [38,48]. Very briefly, a high-*NA* microscope objective focuses a *cw* He-Ne laser onto the sample and collects the fluorescence from the probe molecules. After spectral filtering, a dielectric polarization cube splits the emission into two perpendicular polarization directions, which are detected on separate single-photon-counting photodiodes. For later statistical analysis, the arrival timestamps of every detected photon in each channel are continuously recorded.

The recorded fluorescence intensity in both polarization directions, *I*_||_ and *I_⊥_*, as well as the ratio of these two fluorescence intensities, *ρ* = *I*_||_*/I_⊥_*, exhibit sudden changes, as shown in Figure 10. While changes in the total fluorescence intensity, *I* = *I*_||_ + *I_⊥_*, could be caused by the probe molecule’s photodynamics, such as excursions to the triplet state [1,13,32], or fluctuations in the fluorescence lifetime due to changes in the probe environment [49], changes in the ratio of the fluorescence intensity in the two polarization directions, *ρ* = *I*_||_*/I_⊥_*, indicate reorientations of the probe molecule.

## 4. Analysis Method

The analysis method described here identifies statistically significant changes in the expectation value of the observed intensity ratio. We bin photons from the original photon arrival trajectory with a short bin width of Δ*t* = 5 ms, which is larger than the lengths of typical blinking periods for rhodamine [12,50], corresponding to an average number of about *〈N_photon_〉* ~ 25 photons per bin at typical intensities in our experiments. For each bin we calculate the logarithm of the ratio of the intensity in the two detection channels,

(4) $$\text{log}(\rho )=\text{log}\;\left(\frac{I_{1}}{I_{2}}\right)$$

**Figure 11:**
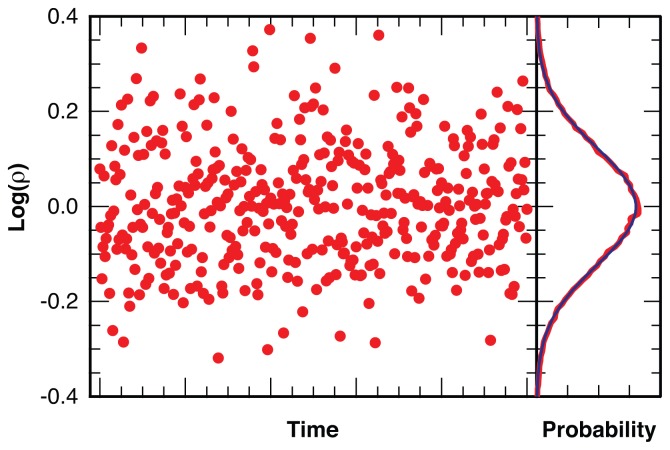
Distribution of the logarithm of the intensity ratio, *ρ* = *I*_||_*/I_⊥_*, for a simulated photon arrival trajectory, binned with *〈N_photon_〉* = 25 photons per bin (red), with fit to a Gaussian distribution (blue).

Stochastic photon counting noise leads to a near-normal distribution [51,52] of log(*ρ*) as illustrated in Figure 11 for simulated photon arrival times in two channels with a constant average intensity ratio of *〈ρ〉* = 1 and *〈N_photon_〉* = 25 photons per bin. At these bin sizes bins without photons are extraordinarily rare and do not require special treatment. The near-normality of the distribution for log(*ρ*), combined with the numerous statistical tools available for the normal distribution, is the reason why we analyze the logarithm of the intensity ratio (Equation 4) instead of the linear dichroism, *I_d_*, (Equation 1) which is traditionally used in fluorescence microscopy.

### 4.1. False Positives

For normally distributed populations the student *t*-test is traditionally used to find statistically significant differences between the means of two samples [53]. We determine the actual threshold values indicating significant differences between the means of two samples (that is, between two consecutive sections of binned intensity ratio values) through random number simulations. To this end we simulate *N* = 100,000 trials of photon arrival time sequences of varying lengths, *L* = 20 to *L* = 5000 points, without a break in the intensity ratio, *ρ*, (testing for false positives). For these simulated photon arrival trajectories we calculate the logarithm of the intensity ratio, log(*ρ*), for bins with *〈N_photon_〉* = 25 photons on average.

At every point, *k*′, with *k*′ = 0*. . . L*, in these intensity ratio sequences we determine the Student’s *t*-test’s *p*-value using standard statistical procedures [53] to test for statistical differences between the sequences before and after the trial point *k*′, {0*, . . ., k*′− 1} and {*k*′*, . . ., L*}, as if *k*′ were an actual break point in the intensity ratio sequence. As the sequences simulated to test for false positives do not contain an actual break point, we save the maximum *p*-value, *p_max_*, found in each of the *N* trials of a given length, *L*. We exclude the *p*-values for the first and last *N_excl_* = 10 potential break points, as those show very large fluctuations due to the small size of one of the two sections tested (Section 2.1). For each length, *L*, we find a threshold value *τ*_1−_*_α_*, such that for a fraction 1−*α* of the *N* trials the maximum *p*-value, *p_max_*, falls below the threshold *τ*_1−_*_α_*, where 1 − *α* is the level of confidence.

### 4.2. Location of Break Points

The simulated example trajectory (with a break point) shown in Figure 4 illustrates the approach used for the analysis of our experimental results. We split a sequence of intensity ratio points into two sections at a trial break point, *k*′, calculate the *p*-value, *p*, for the statistical significance of different means to determine the likelihood, *ℒ*(*k*′) = −log(*p*), for a break at this trial position, *k*′. We repeat the test for all trial break points, *k*′ = *N_excl_* *. . . L* − *N_excl_* in the sequence (again excluding the first and last *N_excl_* = 10 points). We accept the break point, *k*′*_max_*, with the maximum likelihood, *ℒ_max_* = *ℒ*(*k*′*_max_*), as our maximum likelihood estimate, *k̂*, if *ℒ_max_* exceeds the threshold value *τ*_1−_*_α_* for a sequence of length *L* at the confidence level 1 − *α*.

For the analysis of our experimental data we choose a conservative level of confidence of 1−*α* = 99% that in addition has to be surpassed by at least *N_min_* = 10 consecutive trial break points around *k̂* to further guard against false positive identifications caused by additional (non-photon counting) noise in the experiment, for example through frequent blinking of the probe molecule. This safeguard limits the shortest detectable distance between break points, *t_w_*, to about *t_w,min_* *~ N_min_*Δ*t* = 50 ms and raises the detection threshold for break points significantly. The analysis of our experimental data therefore rejects some fraction of the break points with the smallest change in intensity ratio, *ρ_i_/ρ_i_*_+1_. However, for our particular application these rejections are much less of a concern than the inclusion of just a few false positive break points.

### 4.3. False Negatives and Error of Location Estimate

In separate stochastic simulations of photon streams that feature one intensity ratio break in the center, *k* = *L/*2, as illustrated in Figure 4, we determine the fraction of missed break points (false negatives) as a function of the change in intensity ratio, Δ*ρ/ρ*, and the length, *L*, of the sequence probed. We choose a level of confidence of 1 − *α* = 99% plus an additional safeguard of a minimum of *N_min_* = 10 points above the threshold, *τ*_1−_*_α_*, to test the routine under the same conditions as in the analysis of our experimental trajectories. We perform *N* = 10,000 trials for intensity changes of *ρ_i_/ρ_i_*_+1_ = 1.1 to *ρ_i_/ρ_i_*_+1_ = 2.0, with an average number of *〈N_photon_〉* = 25 photons per bin with equal intensity in both channels when averaged over the entire trajectory. For successfully identified break points we determine the distribution of the error, Δ*k̂* = *k̂* − *k*, for the estimated location of the break point, *k̂*, as a function of the length of the sequence, *L*, and the change in the intensity ratio, *ρ_i_/ρ_i_*_+1_, at the break point, *k*.

Furthermore, we determine the difference in the likelihood measure Δ*ℒ_max_* = *ℒ_max_*−*ℒ*(*k*) between the likelihood at the maximum, *ℒ_max_* = *ℒ*(*k̂*) and the likelihood for a break at the actual break point, *ℒ*(*k*). From the distribution of this likelihood difference, Δ*ℒ_max_*, we find a threshold value *τ*′_1−_*_α_* such that for a fraction 1−*α* of the *N* trials Δ*ℒ_max_* falls below *τ*′_1−_*_α_* where 1−*α* signifies the level of confidence for the location error estimate. We use this threshold value *τ*′_1−_*_α_* to find the confidence interval *k*^−^ *. . . k*^+^ for the estimated break point *k̂*, where *k*^−^ and *k*^+^ are the two points to the right and left of *k̂*, respectively, such that *ℒ*(*k*^−^) = *ℒ*(*k*^+^) = *ℒ_max_* − *τ*′_1−_*_α_* (see Figure 4).

### 4.4. Algorithm

**Figure 12:**
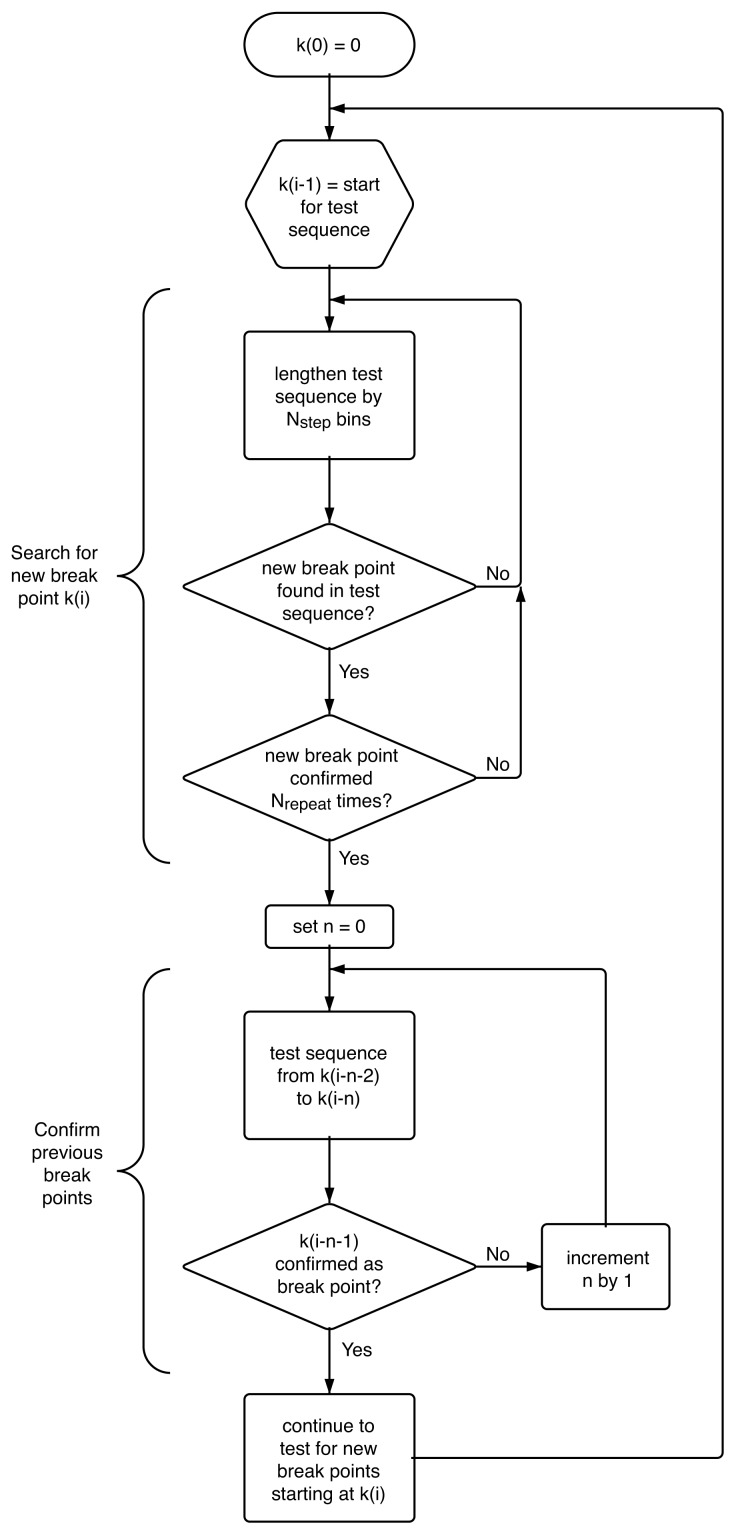
Schematic representation of the algorithm employed to systematically identify a sequence of most likely break points in an experimental trajectory in a self-consistent manner.

The application of the *t*-test as described above can only locate one most probable change point, *k̂*, in a given sequence of points. To find all change points, {*k̂_i_*}, in an experimental trajectory, we systematically test for potential break points in a slowly growing section of the trajectory, starting at the last identified break point, *k̂_i_*_−1_, lengthening the sequence under test by *N_step_* = 5 bins at a time. As an additional safeguard against spurious break points we also require that any probable new break point, *k̂_i_*, is confirmed *N_repeat_* = 2 more times in additional sequences that start with the same previously identified break point, *k̂_i_*_−1_, but are lengthened by an additional *N_step_* bins each time. If the break point is reproducible, we subsequently double-check the previously identified change point *k̂_i_*_−1_ in the sequence bracketed by the two adjacent break points *k̂_i_*_−2_ and (the newly identified) *k̂_i_*. If *k̂_i_*_−1_ is confirmed as the most likely break point location between *k̂_i_*_−2_ and *k̂_i_*, the process continues from break point *k̂_i_* to search for a new break point *k̂_i_*_+1_. However, if the previously identified most likely break point location, *k̂_i_*_−1_, differs from the new location of the break point between *k̂_i_*_−2_ and *k̂_i_*, or if break point *k̂_i_*_−1_ is no longer statistically significant given the new sequence limit *k̂_i_*, break point *k̂_i_*_−1_ is modified accordingly (or eliminated all together) and the confirmation check continues backwards until the sequence {*k̂*_0_ *. . . k̂_i_*} is self-consistent. The algorithm is represented graphically in Figure 12. This approach eventually yields a time sequence of *n* most likely intensity ratio change points, {*k̂_i_*}, with *i* = 0*. . . n*.

We calculate the corresponding intensity ratios, *ρ_i_*, between two identified change points, *k̂_i_* and *k̂_i_*_+1_, directly from the number of photons recorded between these two times in each detection channel. To accelerate the calculation we approximate the *p*-value, *p*, through the following equation that we determined empirically:

(5) $$\text{log}(p)\sim 0.19379t^{2}+0.27472t$$

with

(6) $$t=\frac{\left|{\bar{x}}_{1}-{\bar{x}}_{2}\right|}{\sqrt{\frac{s_{1}^{2}}{N_{1}}+\frac{s_{2}^{2}}{N_{2}}}}$$

where *x* = log(*ρ*), while *χ̄_i_* and *s_i_* are the maximum likelihood estimators for mean and standard deviation, determined for the intensity ratio sequence sections before (*i* = 1) and after (*i* = 2) the trial break point, *k*′. To further improve the speed of the calculation, we pre-calculate cumulative sums for *x* and *x*_2_ for the entire tested sequence, {*ρ*_0_*, . . ., ρ_L_*} to quickly determine averages and standard deviations for the two samples on either side of all possible trial break points *k*′ from differences between the corresponding two elements of the cumulative sums.

### 4.5. Simulation of Photon Sequences With Multiple Break Points

To test the performance, sensitivity, and reliability of the analysis routine algorithm, we simulate the following three types of trajectories with multiple break points: (a) square wave intensities with constant waiting times and constant intensity jumps, varying both parameters independently in separate runs; (b) trajectories with constant waiting time but intensity jumps of random amplitude, varying only the constant waiting time in separate simulation runs; as well as (c) photon sequences that are comparable to experimentally recorded trajectories. The simulation of realistic experimental single molecule trajectories is based on a recently proposed model for the dynamics of glasses [38], which allows us to simulate the waiting times between changes in the fluorescence polarization recorded for the single probe molecule. We simulate the changes in the intensity ratio, *ρ*, at these break points from angular jump trajectories of the single molecule that stem from random walks on a sphere with isotropic exponential jump size distribution. Accounting for the numerical aperture of the microscope objective [54], we subsequently calculate the intensities in the two polarization directions for each orientation, randomly pick photon arrival times with an exponential waiting time distribution, consistent with these intensities in the two detection channels and finally bin the photons as done in the experiment. In these simulations we assume that waiting times and jump sizes are uncorrelated, which is consistent with our experimental results. The purpose of these different simulations is to determine the percentage of identified jumps (sensitivity), the average error in the estimated location of break points (accuracy) of the utilized algorithm (Section 4.4).

## 5. Conclusions

Single molecule spectroscopy is a new and very powerful experimental technique, calling for new analysis methods. The statistical method described in this paper identifies sudden changes in a ratiometric variable, the ratio between two fluorescence intensities, indicating the times of individual dynamical events of the single probe molecule, from the recorded photon arrival times in the two detection channels. This model-free analysis approach provides quantifiable error estimates for the jump times at a chosen level of confidence. Tests on simulated photon arrival trajectories indicate that the analysis method locates all events of significant magnitude with little or no error, and still recovers a large fraction of jumps with small amplitudes. The approach described in this paper is general and can easily be applied to different functional forms of the ratiometric variables to analyze other single molecule experiments, such as *sp*-FRET or spectral diffusion without assuming an underlying kinetic mechanism or a limited number of accessible states. The method is only sensitive to changes in the ratiometric observable and avoids the interference of the frequent and brief single molecule blinking. It therefore fills a gap in the collection of single molecule analysis methods. Moving away from the analysis of single molecule experiments with time correlation functions to a more detailed statistical description using an event perspective affords a higher level of detail accessible in the experiment, bringing us closer to harnessing the full power of both the single molecule and single photon detection technique.

## Figures and Tables

**Table 1:** Best-fit parameters for the empirical fit of Equation 2 to the threshold, *τ*_1−_*_α_,* for statistically significant differences in two sections of intensity ratio change points as determined in random number simulations. *α*: probability of false positive identification, *α_eff_* : probability of false positive identification if the additional safeguard is used that N_min_ = 10 consecutive points have to surpass the threshold value, *τ*_1−α_.

Nominal Confidence, 1 − *α*	Effective Confidence, 1 − *α_eff_*	Amplitude, *A*	scale, *s*	Exponent, *λ*
90%	98%	3.62	0.565	0.285
95%	99%	4.15	0.567	0.249
98%	99.8%	4.98	0.580	0.227
99%	99.95%	5.79	0.608	0.231
99.5%	99.98%	6.80	0.655	0.253

**Table 2:** Parameters for the empirical description of the upper bound for the threshold *τ*′_1−_*_α_,* according to Equation 3, to estimate the confidence interval *k*^−^ *. . . k*^+^, for the estimated break point location, *k̂*, at a level of confidence of 1 − *α*.

confidence, 1 − *α*	small ℒ*_max_/L* constant threshold, *τ*′_0_	amplitude, *A*	exponent, *λ*
68%	1.10	27	1.15
90%	1.95	34	1.12
95%	2.65	37	1.15
98%	3.40	41	1.15
99%	4.20	43	1.15
99.5%	5.20	45	1.18
